# Multistate recursively imputed survival trees for time-to-event data analysis: an application to AIDS and mortality post-HIV infection data

**DOI:** 10.1186/s12874-018-0596-5

**Published:** 2018-11-13

**Authors:** Leili Tapak, Michael R. Kosorok, Majid Sadeghifar, Omid Hamidi

**Affiliations:** 1 Department of Biostatistics, School of Public Health, Modeling of Noncommunicable Diseases Research Center, Hamadan University of Medical Sciences, Hamadan, 65175-4171 Iran; 2 Department of Biostatistics, Department of Statistics and Operations Research, University of North Carolina at Chapel Hill, Chapel Hill, USA; 3 Department of Statistics, Bu-Ali Sina University, Hamadan, Iran; 4 Department of Science, Hamedan University of Technology, Hamedan, 65156 Iran

**Keywords:** HIV/AIDS, Highly active antiretroviral therapy, Random forest, Survival analysis, Recursively imputed survival trees, Cohort studies

## Abstract

**Background:**

This study aimed to introduce recursively imputed survival trees into multistate survival models (MSRIST) to analyze these types of data and to identify the prognostic factors influencing the disease progression in patients with intermediate events. The proposed method is fully nonparametric and can be used for estimating transition probabilities.

**Methods:**

A general algorithm was provided for analyzing multi-state data with a focus on the illness-death and progressive multi-state models. The model considered both beyond Markov and Non-Markov settings. We also proposed a multi-state random survival method (MSRSF) and compared their performance with the classical multi-state Cox model. We applied the proposed method to a dataset related to HIV/AIDS patients based on a retrospective cohort study extracted in Tehran from April 2004 to March 2014 consist of 2473 HIV-infected patients.

**Results:**

The results showed that MSRIST outperformed the classical multistate method using Cox Model and MSRSF in terms of integrated Brier score and concordance index over 500 repetitions. We also identified a set of important risk factors as well as their interactions on different states of HIV and AIDS progression.

**Conclusions:**

There are different strategies for modelling the intermediate event. We adapted two newly developed data mining technique (RSF and RIST) for multistate models (MSRSF and MSRIST) to identify important risk factors in different stages of the diseases. The methods can capture any complex relationship between variables and can be used as a useful tool for identifying important risk factors in different states of this disease.

## Background

There are many biomedical and epidemiological follow-up studies where the subjects may experience events of multiple types. For example, when studying the time to death process in human immunodeficiency virus (HIV)-positive patients, the patients can either experience acquired immunodeficiency syndrome (AIDS) or not before death. One of the main challenges in this research is the need to better understand the prognostic factors affecting the long-term survival in patients to improve their life expectancy. This is usually carried out by fitting separate analyses for each end point as well as for the intermediate events but this is not satisfying because it does not account for the relations between these events [1]. In this regard, using multistate models (MSM), is a natural way to model this kind of complex processes [2].

**Fig. 1:**
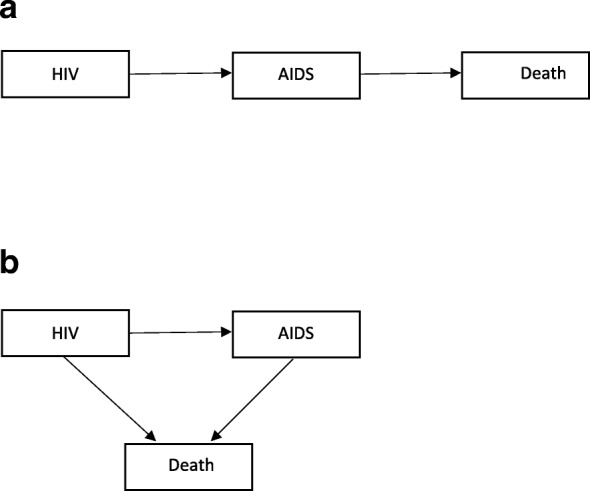
Two simple multistate structures for HIV data; **a**) Progressive multistate model, **b**) Illness-death multistate model

The MSM framework provides a very useful tool to answer a wide range of questions in survival analysis that cannot be answered by classical models [3]. Figure 1 shows two simple but most commonly used cases (progressive and illness-death) of multistate diagrams (for the HIV/AIDS example). Competing risks are another special case of MSMs in which one event precludes the event of interest. In these models, the occurrence of events of interest are considered as transitions from one state to another and where the Markov assumption requires that the transition rates depend only on the current state of the patient and not on the patient’s history [2]. The interest then focuses on predicting the probability that a patient will be in one of the states at some time point after being HIV-infected.

Aalen and Johansen used counting process methods to estimate transition probabilities when there are no covariates [4]. However, in many applications, measured covariates on each individual under study are also available. Therefore, it is often necessary to accommodate the influence of these covariates on transition intensities through a regression model. In this regard, there are a number of models for transition intensities that have been proposed in the literature including parametric models [5–9], semiparametric Markov regression models where transition intensities are modeled by the Cox [10] proportional hazards regression model [11–15], or the Aalen additive hazards regression model [2, 13, 16]. However, most of the time, there are large correlations between covariates as well as non-linear or multivariable relations especially in high dimension settings. This can make the Cox traditional models inefficient and unattractive for variable selection and variable effect estimation [17].

Recently, machine learning techniques have gained increasing attention in many research areas including time-to-event data analysis. Among them, the use of data-driven ensemble methods for covariate selection and prediction in right censored survival data have been suggested by several authors [17–20]. These methods focus on learning a predictive rule which is well-generalized to unobserved data [21]. One of the most popular ensemble learning methods with a broad application in data mining and machine learning techniques is random forests (RF); with, random survival forests (RSF) as its extension to survival data analysis [22]. RSF can automatically handle the issues of traditional methods by combining the ideas of adaptive nearest neighbors and bagging as well as select and rank variables by taking advantage of variable importance measures [22, 23]. Motivated by improving some issues of the Ishwaran’s suggested RSF model such as its requirement of having a minimum predetermined number of (observed failure) events in terminal nodes that consequently makes censored observations hard to use, Zhu and Kosorok [24] developed a procedure to extrapolate as much as possible information contained in censored observations by nonparametric imputation. They proposed to recursively update the censored observations by imputation to the current model-based conditional failure times and to refit the model to these updated data. The final model is then built by repeating this procedure several times. In this method, referred to as recursively imputed survival trees (RIST), the conditional failure times of the censored observations are incorporated into the model fitting procedure. This in turn reduces the prediction error and improves the accuracy of the model [24]. Ensemble learning methods have been used to identify important risk factors in many clinical research settings, with survival outcome, such as time until death, as an endpoint for studying disease processes [25–28]. However, no attempt has been made to exploit them under the multistate models context. In this article, we propose a method which combines RIST [17] with the multistate method, which we call multistate RIST (MSRIST), in the hope of relieving the restrictive assumptions in traditional survival models and improving the predictive power of the resulting model as well as accounting for correlation and interactions among features. We also compare the MSRIST with a multistate version of RSF (MSRSF) as well as with the Cox proportional hazard model in terms of prediction power.

## Methods

### The statistical model

#### Multistate models and terminal node estimators

In this section we give a brief description of multistate models. A multistate model can be described by a stochastic process (*X*(*t*), *t* ∈ *T*)with a finite state space *S* = {1, 2, …, *N*}, where *T* = [0, *τ*] is a time interval (*τ*is the time of the end of the study). The variable t denotes the time since a special event like first diagnosis (for example in HIV patients, it is time from HIV infection), *d* denotes the time of the intermediate event (in HIV positive patients, it is time of developing AIDS). Let *H*_*t*−_ be a history (a *σ*-algebra) generated over the interval [0, t). A history for example consists of information about the patients including transition times from one state to another state. In a multistate framework, the interest is to predict events as well as to discover risk factors for each transition (h → j). Transition probabilities

1 $$ {P}_{hj}\left(s,t\right)=P\left(X(t)=j|X(s)=h,{H}_{s-}\right), $$

for h, j ∈ S, t ∈ T, s ≤ t, or transition intensities

2 $$ {\alpha}_{hj}(t)=\underset{\Delta t\to 0}{\lim}\frac{P_{hj}\left(t,t+\Delta t\right)}{\Delta t}, $$

(the instantaneous hazard of progression to state *j* given current state *h*) can characterize the multistate process completely.

Two commonly used approaches for multistate models are available: (a) the clock forward (in which time *t* is considered since the entrance of the patient to the initial state for all states even for an intermediate event); and (b) the clock reset (in which time *t* in *α*_hj_(t), is considered since the entrance of the patient to state *h*) [29]. There are several model assumptions about the dependency of the transition intensities on time, including being independent of time (constant intensities over time called a time-homogeneous models), depending only on the history of the process via the present state (a Markov model) and depending on the present state (*h*) as well as on the time *T*_*h*_ (the entry time into state h) (a Semi-Markov model) [30].

Regarding two of the approaches, clock forward models can be considered as Markov models and, for clock reset model, the Markov assumption does not hold (because of dependency of the time scale itself on the history through the time since entering to the current state). Nevertheless, by assuming the dependency of the sojourn times on the history of the process only through the present state and the time since entry of that state, a sequence of embedded Markov models can be formulated by the subsequent multistate models (a *semi-Markov* model) [29].

Markov models are commonly used due to their simplicity. In a multistate model, the Markov assumption implies that

3 $$ P\left(X(t)=j|X(s)=h,{H}_{s-}\right)=P\left(X(t)=j|X(s)=h\right), $$

and the transition probabilities are calculated from the intensities by solving the so-called forward Kolmogorov differential equation [31]. Therefore, for the illness-death model, the transition probabilities are explicitly expressed as follows in terms of cumulative intensities between *s* and *t* (i.e.${\mathrm{A}}_{\mathrm{hj}}\left(\mathrm{s},\mathrm{t}\right)={\int}_{\mathrm{s}}^t{\alpha}_{hj}(u) du$):

4 $$ {P}_{11}\left(s,t\right)={e}^{-\left({A}_{12}\left(s,t\right)+{A}_{13}\left(s,t\right)\right)}, $$

5 $$ {p}_{22}\left(s,t\right)={e}^{-{A}_{23}\left(s,t\right)}, $$

6 $$ {P}_{12}\left(s,t\right)={\int}_s^t{P}_{11}\left(s,t\right){\alpha}_{12}(u){P}_{22}\left(u,t\right) du. $$

These probabilities can be estimated through the non-parametric model (e.g. the Aalen-Johansen estimator) [32]. For example the Nelson-Aalen estimator of cumulative hazard for h → j transition at *t* is

7 $$ {\widehat{\mathrm{A}}}_{\mathrm{hj}}\left(s,t\right)=\sum \limits_{s\le t}\frac{\Delta {N}_{hj}(s)}{Y_h(s)},h\ne j, $$

where *ΔN*_*hj*_(*s*) ≔ *N*_*hj*_(*s*) − *N*_*hj*_(*s*^−^) is the number of h → j transitions observed exactly at time *s* and *Y*_*h*_(*s*)is the number of individuals at risk in state *h* just prior to time *s*. Moreover, the elements of the transition probability matrix can be estimated as follows [2]:

8 $$ \widehat{P}\left(s,t\right)=\prod \limits_{\left(s,t\right\}}\left\{I+d\widehat{A}(u)\right\}. $$

On the other hand, in a semi-Markov model, transition probabilities and intensities are as follows

9 $$ {P}_{hj}\left(s,t,{T}_h\right)=P\left(X(t)=j|X(s)=h,{T}_h\right) $$

and

10 $$ {\alpha}_{hj}\left(t,{T}_h\right)=\underset{\Delta t\to 0}{\lim}\frac{P_{hj}\left(t,t+\Delta t,{T}_h\right)}{\Delta t}, $$

which are not fixed (because they depend on the random quantity of *T*_*h*_), and there are no Kolmogorov equations for them. However, in general it is still possible to derive transition probabilities from transition intensities even though the theory is more complex [33].

There are different approaches for the estimation of the transition hazards for censored data based on common regression models. For example, in a so-called separate approach, all transition hazards are modeled separately. Here, we use the separate approach to build our multistate trees.

#### Recursively imputed survival trees for multistate model

MSRIST consists of the following steps as suggested by [24] in a single point survival analysis setting:

1. **Multistate tree model fitting:** fit the number of *M* extremely randomized multistate trees (ERMTs) to the initial training set (instead of bootstrapped samples). To this end *M* extremely randomized multistate trees (one tree for each transition) for the raw training dataset are generated under the following settings: for each split for the h → j transition, *K* candidate covariates (along with random split points for each) are randomly selected from (*p)* covariates. Then the best split (that leads to the most distinct daughter nodes) will be determined for each transition using the log-rank test; and for each transition the splitting process will be continued until a terminal node contains less than nmin > 0 observed events.
2. **Conditional transition distribution:** A conditional survival distribution is calculated for each censored observation.
3. **One-step imputation for censored observations:** All censored data in the raw training dataset will be replaced (with a correctly estimated probability) by one of two types of observations: either an observed failure event with Y < τ, or a censored observation with Y = τ.
4. **Refit imputed dataset and further calculation:** *M* independent imputed datasets are generated according to 3, and one multistate tree is fitted for each of them using 1(a) and 1(b).
5. **Final prediction:** Steps 2–4 are recursively repeated a specified number of times before final predictions are calculated.

#### Random survival forests algorithm for multistate models

We will focus on models that satisfy the Markov assumption, but results are applicable to non-Markov models as well by considering *d* and *t-d* as covariates in the forests as suggested by [22]. The details of the algorithm are as follows:

1. Draw *B* bootstrap samples from the original data while excluding about 37% of the data in each bootstrap sample (out-of-bag or OOB data);
2. Grow a multistate survival tree for each bootstrap sample based on randomly selected *K ≤ p* candidate variables at each node of the tree. The candidate variable, used to split each node for the h → j transition, is the one that maximizes a splitting rule (e.g. using a log-rank test);
3. Grow the trees to full size under the constraint that a terminal node should have no less than n_0_ *>* 0 unique cases for each transition;
4. Calculate ${\left({\widehat{P}}_{hj,b},{\widehat{A}}_{hj,b}\right)}_{\mathrm{h},\mathrm{j}\in \mathrm{S}}$ for each tree, *b*;
5. Take average of each estimator over the B trees.

#### Prediction performance

To evaluate prediction performance of the model, the prediction error can be estimated through the integrated Brier score (BS), the squared difference between actual and predicted outcome. The Brier prediction error for state *h* is given by [34]:

11 $$ {PE}_B^h(s)=E\left[{\left(I\left\{X(s)=h\right\}-{\widehat{\pi}}_h\left(s|z\right)\right)}^2\right], $$

which is estimated by

12 $$ P{\widehat{E}}_B^h(s)=\frac{1}{n}\sum \limits_{i-l}^n\left[{\left(I\left\{{x}^i(s)=h\right\}-{\widehat{\pi}}_h^{(n)}\left(s|{z}^i\right)\right)}^2\right] $$

in the case of a complete observation, where *x*^*i*^(*s*) for any time point can be computed by ${x}^i(s)=\sum \limits_{m=0}^{M-1}I\left\{{t}_m^i\le s<{t}_{m+1}^i\right\}{x}^i\left({t}_m^i\right)$, ${t}_0^i=0,{t}_1^i,\dots, {t}_M^i$are the transition times for each individual, ${x}^i\left({t}_m^i\right),m=0,\dots, M,$ are the state occupied at these times and ${\widehat{\pi}}_h$is a prediction for the transition probability. For the case of right censoring, the sample contains${\left\{\Big({\tilde{t}}_m^i,{\tilde{x}}^i\left({\tilde{t}}_m^i\right)\Big)\right\}}_{m=1}^M$,$\left({\tilde{t}}^i,{\tilde{x}}^i\left({\tilde{t}}_m^i\right)\right)$,*δ*^*i*^and *z*^*i*^for each individual. Then, using the inverse probability of censoring weights (IPCW) technique, the estimator becomes as

13 $$ P{\widehat{E}}_B^h(s)=\frac{1}{n}\sum \limits_{i=1}^n\left[w\left(s,{\tilde{t}}^i,{\tilde{x}}^i(s),{\widehat{G}}^{(n)},{z}^i\right){\left(I\left\{{\tilde{x}}^i(s)=h\right\}-{\widehat{\pi}}_h^{(n)}\left(s|{z}^i\right)\right)}^2\right], $$

where

14 $$ w\left(s,{\tilde{t}}^i,{\tilde{x}}^i(s),{\widehat{G}}^{(n)},{z}^i\right)=\frac{I\left\{{\tilde{t}}^i\le s,{\tilde{x}}^i(s)\ne 0\right\}}{{\widehat{G}}^{(n)}\left({\tilde{t}}^i-|{z}^i\right)}+\frac{I\left\{{\tilde{t}}^i>s\right\}}{{\widehat{G}}^{(n)}\left(s|{z}^i\right)}. $$

The BS measures the mean squared difference between the predicted probabilities for a possible outcomes for a subject and the observed outcome. So, it is the mean square error for a prediction and has been widely used in survival data context. The smaller the IBS, the better the predictions is returned by a model.

#### Cindex

Another criteria that was used in this study was concordance index (*Cindex*) for survival data. *Cindex* is a measure of the discriminative power of a model. In each state, two patients (a pair) in survival analysis are concordant if the predicted risk of the interesting event based on the model is greater for the patient who experiences the event at an earlier time point. The *Cindex* is then calculated by using the frequency of concordant pairs among all pairs of subjects. *Cindex* takes its values between 0 and 1 and the greater the values the better the discriminative power [35].

#### Variable importance

Importance of the variables was assessed by variable importance criterion (VIMP). To calculate the VIMPs, the sum of the decrease in prediction error is considered when a split by a special variable is made. Therefore, following the structure proposed by Ishwaran et al. [22] the VIMP was calculated as follows: a) the data was randomly divided into train and test sets; b) a MSRIST was created using the data in the training set; c) for a variable say *x*, new cases (in the test set) were dropped down the tree and they were assigned randomly to a daughter node whenever a split for x is encountered for each state; d) for each state the cumulative hazard function is calculated from all trees and averaged; e) the VIMP for x is calculated by subtracting the prediction error of the original ensemble and the new ensemble that is obtained by random allocation for *x*.

### Application

#### Data source

This study utilized a data set corresponding to a registry-based retrospective cohort study conducted in Tehran, Iran, from April 2004 to March 2014. The population in the present study involved people who were HIV-infected and who had a medical record in either Behavioral Diseases Counseling Centers in Tehran (Imam Khomeini or Zamzam Centers). A person who had been infected with HIV was regarded as an HIV-positive case, regardless of the clinical stage confirmed by laboratory criteria according to the country definitions and requirements [36]. An HIV case, in the Islamic Republic of Iran, was an individual who had two positive sequential enzyme-linked immunosorbent assay (ELISA) tests for HIV antibody followed and confirmed by a western blot test [37] and an AIDS case was defined as a presumptive (definitive) diagnosis of stage 4 conditions and/or CD4 count less than 200 per mm^3^ of blood in an HIV-infected subject [36].

#### Study variables and outcomes

The following variables were assessed for prognostic value using a checklist of items, developed according to the information documented in the medical records such as: demographic information (age, sex, marital status, and educational level), behavioral information (drug abuse, smoking, and being in prison), baseline CD4 cell count (cells/mm3), highly active antiretroviral therapy (HAART or ART, a combination of several antiretroviral medicines (which is believed to be more effective than using just one medicine (monotherapy)) used to suppress HIV viral replication and to slow down the progression of HIV disease [38, 39]. The combination usually includes several drugs such as two nucleoside reverse transcriptase inhibitors (NRTIs) (e.g., Abacavir, Emtricitabine, and Tenofovir), a protease inhibitor (PI) (e.g., Atazanavir, Darunavir, and Ritonavir)), co-infection with TB, and causes of death (according to the information documented in the medical records).

There were two primary endpoints: 1) AIDS development; and 2) AIDS-related death. So, the outcomes of interest was the duration of time from the HIV diagnosis date to AIDS progression (HIV → AIDS transition) and from AIDS to AIDS-related death (AIDS →Death transition). Censoring included those patients who were lost to follow up and those who were alive at the end of the study period.

#### Data description

**Fig. 2:**
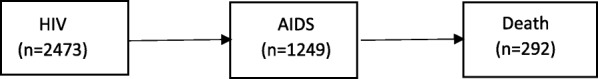
The structure of the application and corresponding sample sizes

There were 25 ineligible patients and 21 patients with a medical record in both centers among 2519 identified patients in the present study. We considered the data from the 2473 patients (1937 men and 536 women) whose information was appropriate for the analysis. The mean (standard deviation) age of the patients was 34.01 (10.43) years, ranged from infancy to 74 years. Table 1 illustrates the characteristics of the study population. There were 1249 patients who developed AIDS, where 292 out of them died from AIDS-related causes (Fig. 2). Other patients, who were alive or lost to follow up at the end of the study, were considered to be censored.

**Table 1:** Characteristics of the study population infected with the HIV virus

Variables	Number	Percent
Gender		
Female	505	22.45
Male	1744	77.55
Age group (year)		
1–24	260	11.60
25–44	1639	73.10
45–74	343	15.29
Marital status		
Single	874	40.37
Married	852	39.35
Divorced	330	15.24
Widow	109	5.03
Education level		
High (academic)	147	7.34
Low (school)	1856	92.66
Being in prison		
No	899	39.97
Yes	1350	60.03
Smoker		
No	933	46.91
Yes	1056	53.09
Drug abuse		
No	1119	49.75
Yes	1130	50.24
Tuberculosis infection		
No	2012	89.46
Yes	237	10.54
Antiretroviral therapy		
No	1315	58.47
Yes	934	41.53
Baseline CD4 count (cells/mm3)		
500+	417	21.55
351–500	296	15.30
201–350	415	21.45
0–200	807	41.70

The majority of the HIV-positive patients were male (77.55%) and aged 25–44 years (73.1%), single (40.37%), less-educated (*92.66%*). In addition, 53.09% of them were smokers, 50.24% were drug abusers and about 60% of them had a history of being in prison. Also, about 10.54% of the patients co-infected with TB and 41.53% of them had used antiretroviral therapy.

#### Implementation

To implement the two tree-based methods of MSRSF and MSRIST, the shared tuning parameters like the minimum number of events in terminal nodes were fixed (to make fair comparisons). Therefore, as suggested by [22], the integer part of the square root of the number of covariates was used for *K* (the number of variables at each splitting). Moreover, the minimal number of events in each terminal node for all transitions was set to 6. For MSRSF, the forest consisted of 1000 trees. The log-rank splitting rule was used for MSRSF. For the MSRIST model, 50 trees (*M*) were used in each of five imputation cycles. We also fitted the Cox proportional hazards (PH) model with the Lasso penalty where the tuning parameter was determined using 10-fold cross-validation using the “Penalized” package in R [40]. We randomly divided the data set into training and testing data sets and repeated the methods 500 times. The models were fitted to the training sets and evaluation criteria were calculated over the test sets.

## Results

The results of fitting MSRSF, MSRIST and the Cox Model are presented for both transitions HIV → AIDS and AIDS →Death in Table 2. As there was no deaths from causes not related to AIDS, the structure of the data was considered as a progressive multistate model.

**Table 2:** Integrated Brier score (IBS) and Cindex values for three methods (Cox, MSRIST and MSRSF) over 500 repetitions

	Transition					
	HIV ➔ AIDS		AIDS ➔ Death (Markov Assumption)		AIDS ➔ Death (Non-Markov assumption)	
Method	IBS	Cindex	IBS	Cindex	IBS	Cindex
Cox	0.126 (0.008)	0.747 (0.008)	0.143 (0.013)	0.638 (0.033)	0.139 (0.012)	0.637 (0.034)
MSRSF	0.123 (0.009)	0.768 (0.009)	0.110 (0.009)	0.703 (0.035)	0.111 (0.010)	0.702 (0.036)
MSRIST	0.113 (0.003)	0.802 (0.004)	0.099 (0.004)	0.762 (0.024)	0.101 (0.006)	0.759 (0.025)

As shown, the MSRIST outperformed both MSRSF and the Cox models in terms of both criteria (IBS and Cindex). So for the HIV → AIDS transition, the mean (standard deviation) of IBS and Cindex related to MSRIST were 0.113 (0.003) and 0.802 (0.004) respectively. In addition for AIDS →Death transition, the mean (standard deviation) of IBS and Cindex related to MSRIST were 0.099 (0.004) and 0.762 (0.004) respectively. As suggested by [1], we considered an additional situation where sojourn times were considered as covariates to investigate their effect on the survival. However, there was no meaningful change in evaluation criteria.

**Fig. 3:**
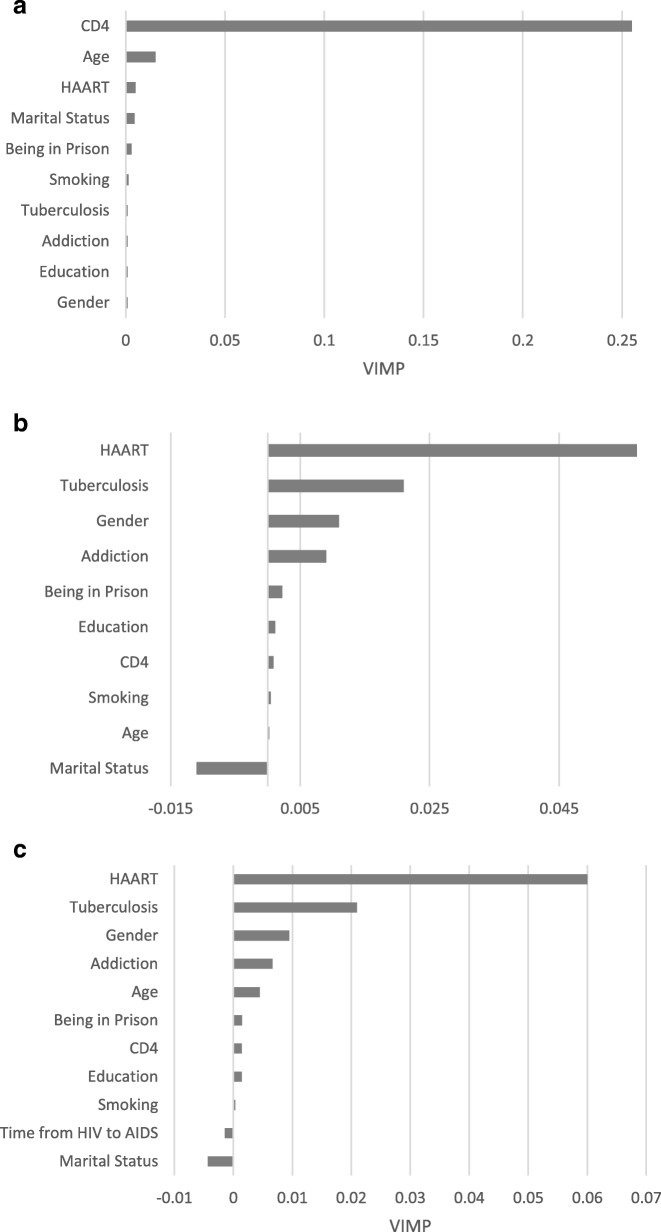
Variable importance for transitions from (**a**) HIV to AIDS, (**b**) AIDS to Death (under a Markov assumption) and (**c**) AIDS to Death (under a Non-Markov assumption)

The values of the VIMP were calculated. The most predictive variables for the present study were defined as those whose VIMP (averaged over the forest) were greater. As the MSRIST led to better predictive power, we only calculated the VIMP for this model. Figure 3(a) and (b), depicts all variables and plots their VIMP for HIV → AIDS and AIDS →Death transitions, respectively. According to the figure the three top most important variables for time from HIV infection to AIDS progression which were baseline CD4 cells count, age and antiretroviral therapy respectively. In addition, for the transition from AIDS to death the three top most important variables were antiretroviral therapy, TB and Gender, respectively. The VIMP of the variables for the setting in which sojourn times were considered as covariates were shown in Fig. 3(c). As seen, the time since HIV → AIDS did not play an important role as a covariate in modeling AIDS →Death transition.

**Fig. 4:**
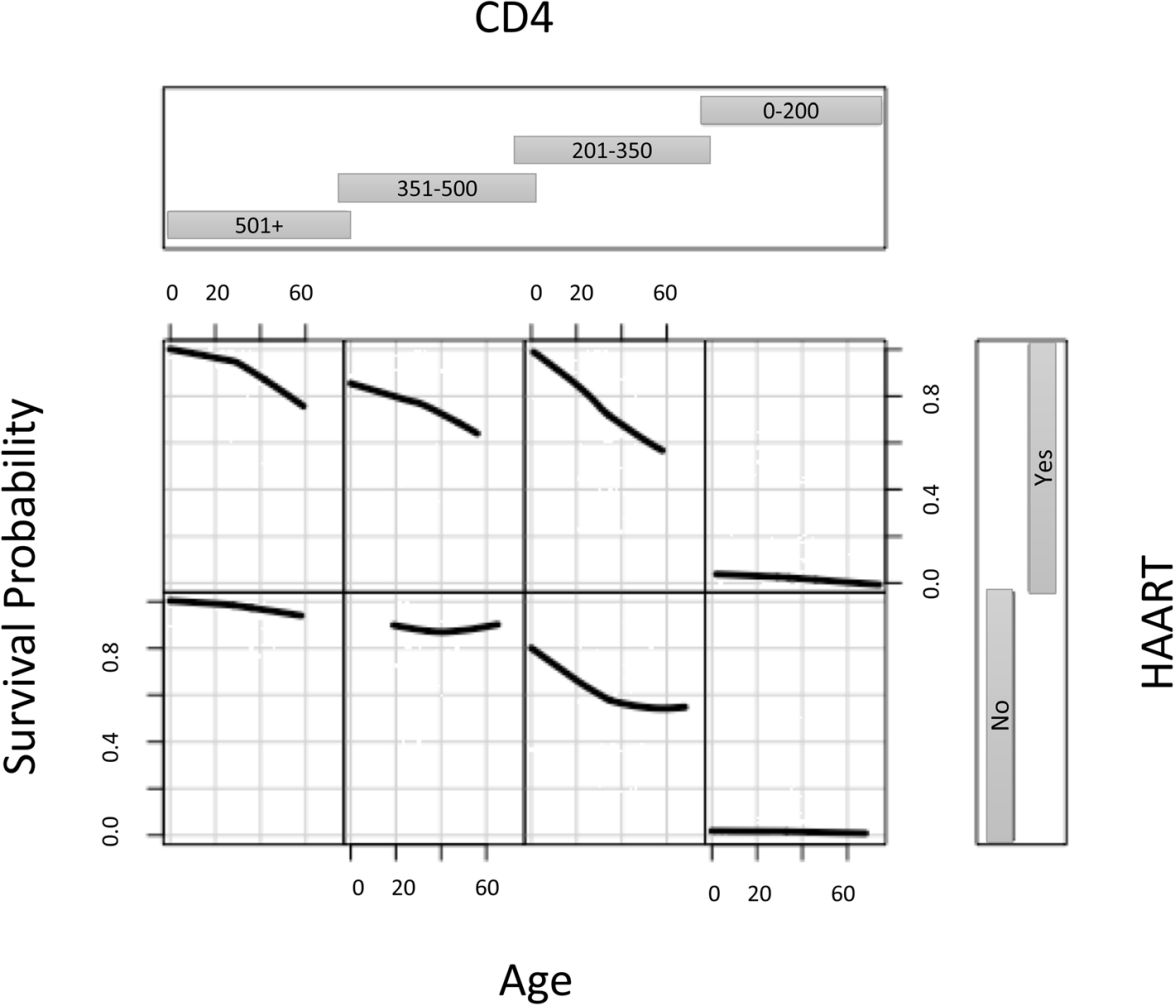
MSRIST estimated three-year survival of the HIV-infected patients for progression to AIDS as a function of HAART, age and baseline CD4 count. Smoothed curves are loess curves of the estimated survival for each individual

**Fig. 5:**
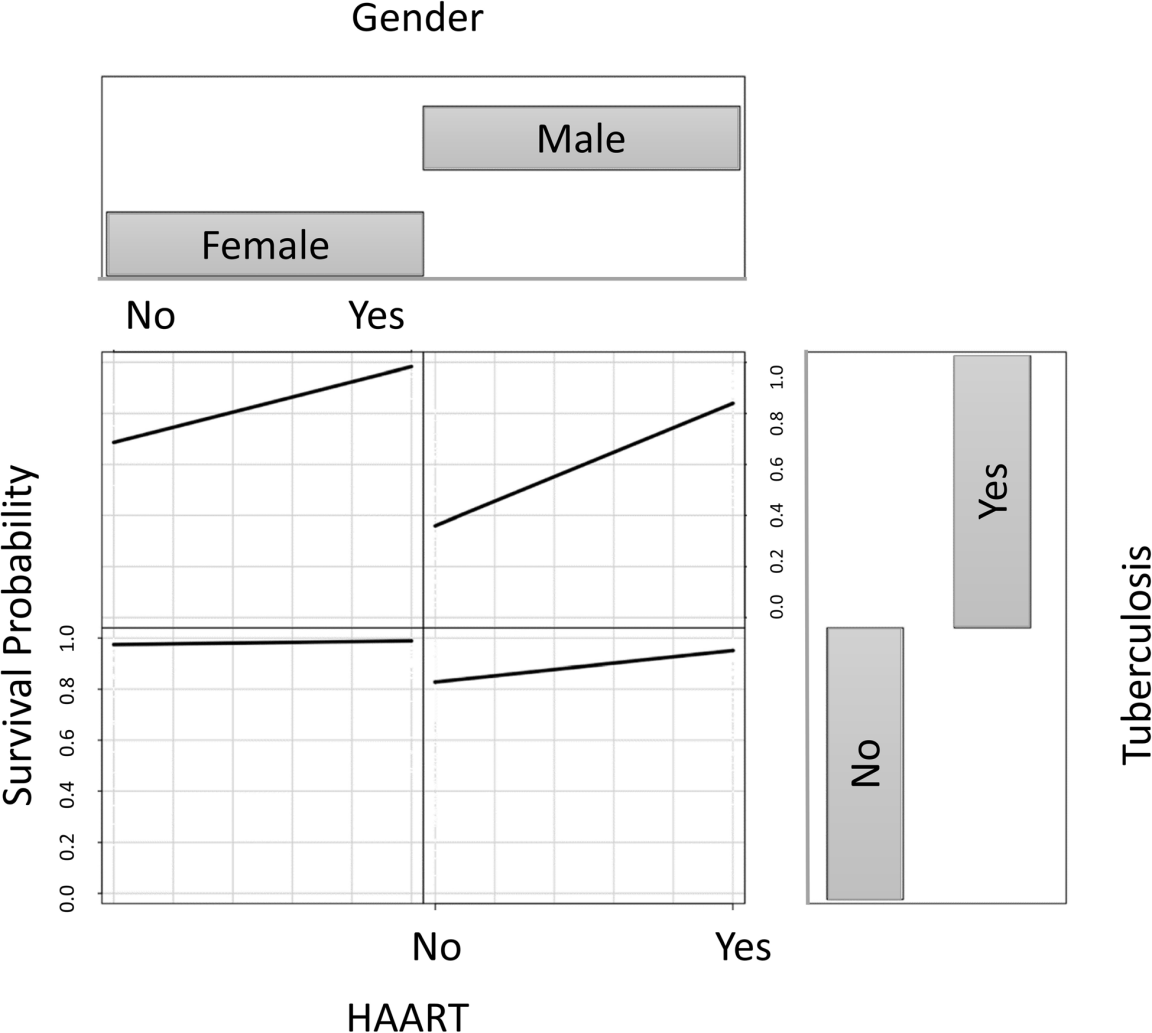
MSRIST estimated three-year survival of the AIDS-progressed patients for progression from AIDS to death as a function of TB, HAART and gender. Smoothed curves are loess curves of the estimated survival for each individual

Figures 4 and 5 display the interaction between the three most important variables for both transitions using the MSRIST model and 3 year predicted survival. For the HIV → AIDS transition, patients with the CD4 count smaller than 200 and not using HAART have the worst survival. Survival did not change much for those with CD4 count > 500. According to Fig. 5, the worst survival (for AIDS →Death) is related to men who have TB and are not using HAART.

## Discussion

There are so many diseases which include intermediate events. Multistate models provide an evolving method in survival analyses. In this study, a new multistate survival data analysis was proposed by introducing RSF and RIST methods into the multistate modeling framework. Application of the MSRIST approach provides an alternative way to build a risk prediction model while preventing the imposition of parametric or semi-parametric constraints on the underlying distributions. Moreover, this method provides a way to automatically address high-level interactions and higher-order terms in variables for different transitions of the disease process while allowing accurate prediction [41].

We applied MSRIST to identify important prognostic factors affecting duration of time to two states in HIV-infected patients (HIV → AIDS and AIDS →Death; a progressive multistate model). Several risk factors strongly associated with survival time of transitions to both states (AIDS and Death) were identified (HIV → AIDS and AIDS→Death). Among them, MSRIST identified baseline CD4 count, age and antiretroviral therapy as the top three most important predictors of survival for the duration of time from HIV diagnosis to AIDS progression and antiretroviral therapy, TB and gender for the duration of time from AIDS diagnosis to death.

It was shown that the baseline CD4 count is the top first important predictor of progression to AIDS. The results suggest that predicted 3-year survival was dramatically diminished for the patients who had a CD4 cell count less than 200 cells/mm3 compared to other levels. Several epidemiological studies have shown an increase in the risk of HIV/TB coinfection as the CD4 cell count decreases [39, 42, 43]. High levels of CD4 cell count (over 500 cells/mm3) reduces TB-related mortality among HIV-positive people as well as those not co-infected with TB and therefore it plays an important role in the incidence of HIV/TB co-infection [44]. Age was the second most important variable for the HIV → AIDS transition. According to epidemiological studies, patients aged 50 years or over are at a higher risk of progression to AIDS compared to younger patients (based on the Cox model) [39, 45–47].

A leading preventable cause of death among people living with HIV is TB [48]. According to our findings, time to transitions from AIDS to death for an HIV-infected patient was highly associated with TB co-infection and the results showed that it plays an important role in AIDS-related deaths. This prognostic factor was the second top most important variable for progression from AIDS to AIDS-related deaths. The epidemiological studies confirmed this finding [38, 39, 49]. Therefore, the importance of treatment of TB in HIV infected people is revealed by this evidence. It was also shown that HAART plays an important role in survival of HIV-infected patients in both transitions. This effect was not shown in the traditional Cox model in the previously published paper on this dataset [39]. According to the results, using antiretroviral therapy increases 3-year survival of the patients for AIDS progression and AIDS-related deaths considerably. It was the third top most important variable for AIDS progression and the top most important variable for progression from AIDS to death. This finding is in agreement with several studies [41, 50, 51].

The present study was conducted based on a large data-set and the results can be generalized to the Iranian HIV-infected population. The effect of several predictors on AIDS progression and AIDS-related deaths, in a high-middle-income country, was evident [39]. This kind of information may help establish intervention measures to suppress the progression of HIV to AIDS and to reduce the risk of death among HIV-positive patients [39].

We adapted two newly developed data mining technique (RSF and RIST) for multistate models (MSRSF and MSRIST) to identify important risk factors in two different stages of the disease. Several studies confirmed RSF’s promising performance in survival analysis compared with traditional Cox proportional hazards model [26, 27, 41]. Zhu and Kosorok [24] also showed that RIST outperforms RSF and the Cox model in classical survival data settings (with just one event of interest), and they have provided a detailed discussion about why RIST works. In the present study, it was also shown that the proposed method based on RIST works in multistate data analysis as well. The usual multistate regression methods are dependent on the Markov assumption which can be restrictive. In the proposed methodology, handling the non-Markov setting is straightforward. To this end, one should consider transition times as covariates which provide researchers with the ability to account for time-varying effects of other covariates. Taking into account the transition times may also provide a test to check the Markov assumption. If the value of the variable importance is large, it could be concluded that the Markov assumption does not hold. The other advantage of the proposed method is that it can take into account nonlinear effects of the covariates in each state as well as high order interaction between them. Therefore, a flexible functional form for the covariates is considered which can be easily uncover highly complex interrelationships between variables [23, 52]. The proposed method can be easily used in complex multistate data settings. MSRIST preserves information of the censored observations through computing the conditional survival function and improves the model prediction by using the updated conditional failure information. The main advantage of the MSRIST model is its tree-based model building. This is because of the fact that larger trees are grown by using the whole training data instead of using bootstrapped samples and there are more observed events (by imputing the survival time of censored observations) to create deeper trees. The overfitting issue is also avoided by the diversity established through randomness in imputation steps [24]. Moreover, the MSRIST is more nonparametric, requiring weaker model assumptions. In spite of these benefits for the MSRIST model, there are some drawbacks for the presented model. For example, it is more variable than parametric approaches and interpretation of the model can be challenging. It is suggested that the performance of the proposed method is investigated in other datasets.

### Future Research

Recently, joint modeling of a longitudinal response process and a time-to-event outcome has gained considerable attention and it is an open research area. A common objective in these studies is to characterize the relationship between two outcomes simultaneously. A potential promising extension of the model proposed here is to introduce the RIST/MSRIST into joint modeling.

## Conclusions

We proposed a new strategy for multi-state frame work modelling and investigated the performance of the method for modeling the intermediate event. We showed that this new method outperformed the classical Cox regression model as well as our other proposed method based on random survival forest. Data mining techniques can be used as a useful tool in the multi-state modeling context. Further investigations are needed with other data sets.

## Abbreviations

*AIDS*: Acquired immunodeficiency syndrome
Cindex: Concordance index
ERMT: Extremely randomized multistate trees
HAART/ART: Highly active antiretroviral therapy
*HIV*: Human immunodeficiency virus
MSRIST: Multistate recursively imputed survival trees
MSRSF: Multi-state random survival Forest
NRTIs: Nucleoside reverse transcriptase inhibitors
OOB: Out-of-bag
RIST: Recursively imputed survival trees
RSF: Random survival Forest
TB: Tubercolosis
